# Transforming South Africa’s school nutrition programme for educational success: A review on challenges and prospects

**DOI:** 10.1177/22799036251380781

**Published:** 2025-11-10

**Authors:** Adrino Mazenda, Aboleleng Monedi, Ni Putu Wulan Purnama Sari, Chamunorwa Huni

**Affiliations:** 1 School of Public Management and Administration, University of Pretoria, Hatfield, South Africa; 2 Faculty of Nursing, Widya Mandala Surabaya Catholic University, East Java, Indonesia; 3 School of Government, University of Western Cape, Cape Town, South Africa

**Keywords:** national school nutrition programme, total quality management, educational outcomes, South Africa

## Abstract

**Introduction:**

South Africa’s National School Nutrition Programme (NSNP) was created in 1994 to reduce poverty and unemployment and improve children’s learning. It aims to aid disadvantaged children and strengthen poor communities, especially in rural areas.

**Objectives:**

The study examined challenges in implementing Total Quality Management (TQM) in the South African National School Nutrition Programme.

**Methods:**

A qualitative case study design was used based on document analysis extracted through the Preferred Reporting Items for Systematic Reviews and Meta-Analyses (PRISMA) method. The documents were analysed using thematic analysis.

**Results:**

Key TQM challenges affecting the implementation of the NSNP include *Customer focus*—lack of funding from the Department of Basic Education to accommodate increased enrolment; Employee involvement—role ambiguity due to lack of communication, and at times, the roles of the NSNP players are not clearly defined; *Process-centric approaches*—lack of school-level infrastructure to store and cook for students, *Continuous improvement and training*—lack of education curriculum on nutrition education and *Relationship management*—lack of coordination of the stakeholders on funding, political landscape, nutrition policies, community involvement, programme adaptability.

**Conclusion:**

Effective monitoring systems should be established to guarantee that students receive the necessary quality and quantity of food. These systems should also oversee food supply, storage, preparation, and hygiene. Additionally, the current targeting system, the quintile system, requires evaluation. This should involve assessing the school’s location, available resources, and individual students’ social context.

## Introduction

The National School Nutrition Programme (NSNP) of South Africa, established in 1994, is committed to achieving Sustainable Development Goal (SDG), Zero Hunger. This programme aims to combat malnutrition, enhance educational achievement, and foster child growth and development.^1^ It embodies principles of equity and inclusivity, ensuring every child has access to nutritious meals regardless of their socio-economic background.^2^ The NSNP operates in over 21,000 schools, 95% are public institutions.^1^ More than a third of enrolled students come from households with incomes below the poverty line. In November 2023, the average monthly cost of a basic nutritious diet for a child was R946.98, in South African Rand (R), or US$0.53. This indicates that the R510 (US$29) Child Support Grant is insufficient to cover the cost of a nutritious diet and falls below the upper-bound poverty line of Rands 1558 (US$88).^3^

The NSNP is a community-driven initiative supported by the government and residents. It relies on the voluntary participation of parents and community members, who assist with food handling at a ratio of 1:200, maintain school food gardens, donate kitchen facilities, protect school resources, and supply cooking, eating, and gardening equipment.^4^ This active participation is vital to the programme’s success, as it helps every community member feel valued and integral to the initiative. School principals and nutrition coordinators are critical in managing the NSNP and are engaged in its Total Quality Management (TQM).^5^

TQM is an ongoing process focussed on detecting, reducing, or eliminating errors.^6^ It streamlines management, enhances the customer experience, and ensures proper employee training.^7^ In school feeding schemes, TQM involves monitoring products, services, and current procedures to maximise productivity while minimising waste and considering school feedback to improve the programme.^8^ This process includes ensuring that food deliveries are timely, checking expiration dates, and determining the daily quantity needed based on each school’s requirements, as communicated to the NSNP coordinators^4^ According to Mawela and van den Berg, ‘the main goal of the NSNP is to improve students’ learning abilities by providing them with healthy meals at school and ensuring that their health and safety are not compromised in class so they can learn effectively’.^2^ Additionally, the NSNP strives to enhance students’ punctuality, regular school attendance, focus, and overall well-being.^9^ However, Berejena and Kleynhans found that only one of the three NSNP objectives had been met.^4^ Furthermore, efforts to improve food production and nutrition education are not prioritised, and there is inadequate community involvement and inefficient programme administration, which is essential for creating a healthy learning environment.^2^ Previous studies have evaluated challenges in the implementation of NSNP.^10–12^ However, there is a lack of literature on examining challenges in implementing TQM in South Africa’s NSNP. Research is needed to understand existing knowledge gaps to inform policy prescriptions to effectively enhance the NSNP implementation in South Africa. This study aims to address the question:

*What are the challenges in implementing TQM in the South African National School Nutrition Programme?*

## Literature review

*The Ecological Systems Theory*, developed by Urie Bronfenbrenner, emphasises the influence of multiple environmental systems on an individual’s development.^13,14^ In the context of school nutrition programmes, it considers how various levels (microsystem, mesosystem, exosystem, macrosystem) influence a student’s eating habits and nutritional outcome.^15^ Understanding these influences can assist schools in designing more effective programmes that support healthier eating behaviours among students.

*The microsystem level* refers to the school environment, encompassing school facilities, classrooms, dining areas, and interactions with teachers, peers, and staff. These factors can influence students’ eating behaviours.^16^ For example, a welcoming dining area can support healthy eating habits, while positive engagement with staff can enhance a supportive atmosphere. Availability, the availability of nutritious food options within the school, can further impact students’ food choices. Burnett highlighted the role of interrelated systems and demographic and developmental influences in shaping programme participation and involvement.^17^ Recognising these elements is crucial for schools to develop an inclusive and effective programmes that resonate with all students.

*The mesosystem level* entails coordinating schools, parents, and community organisations to implement support services consistently. Scholars such as Wittstock et al. underscore the importance of recognising community realities in developing effective programmes that facilitate negotiations between the education department and a South African primary school.^2,18^

The *exosystem* encompasses government policies and regulations contributing to the programme.^16^ Economic factors, like funding availability and community socioeconomic conditions, also play a role in programme sustainability and reach.^19^ Mansvelt et al. extended this application to the issue of food insecurity in South African higher education institutions, emphasising the need for holistic response strategies.^20^

The *macrosystem* refers to broader cultural, societal, and political influences that shape the implementation and effectiveness of the NSNP.^13,14^ Understanding these factors assists in designing interventions that are culturally sensitive and responsive to the needs of diverse communities.^1^ By considering the unique contexts of each community, we can develop more effective strategies to improve nutrition and health outcomes for all students.

The *chronosystem* encompasses how the NSNP and its impact evolve.^16^ Changes in government policies, economic conditions, community demographics, and societal attitudes towards nutrition all influence the programme’s long-term effectiveness and sustainability.^13,14^ Tshisikhawe et al. reiterate that monitoring and adapting the programme in response to these changes are essential for ensuring continued student benefits.^1^ The empirical literature on how other countries implemented the TQM components in their school nutrition programmes draws on various country studies and specific TQM components. One crucial aspect that emerges from this literature is the emphasis on the customer focus component of TQM, which highlights the importance of placing the learner at the centre of decision-making. Several studies argue that insufficient kitchen facilities are failing to satisfy the customers who are students. Exploring the long-term sustainability of school-based nutrition and food programmes: What works, where and why? *Health Promotion Journal of Australia*. https://doi.org/10.1002/hpja.847). A study in Brazil by Alves Da Silva et al. indicates that insufficient kitchen facilities affect meal quality and student satisfaction, reducing community participation in school feeding programmes.^21^ Another study in China supports that obsolete kitchen facilities affect the preparation of nutritious meals, negatively affecting students’ health outcomes.^22^ They found that improving kitchen facilities is crucial for enhancing the effectiveness of school nutrition programmes, especially the smart kitchen technology, which is useful for improving food quality.^22^ Findings from other studies suggested that limited resources concerning school kitchens have caused schools to depend on less nutritious food options, undermining student health initiatives.^10^ Community-sharing models may enhance access to kitchen facilities, thus improving overall service quality.^10^

Implementing *process-centric approaches* in TQM is confronted with different challenges, and solutions are needed. In the United States, the common challenge is bureaucratic and inconsistent funding, hindering effective programme implementation.^23^ Another study in Brazil identified challenges in establishing standardised food portions to guarantee quality and safety in food supply chains.^24^ In Zimbabwe, logistical issues and limited funding complicate the process-centric approaches.^25^ Mu et al. suggested solutions include successfully integrating technology to monitor food quality and nutritional standards, illustrating how data-driven methods can progress programme effectiveness.^26^ Velten et al. indicated that community involvement and partnerships with local farmers effectively overcome these barriers and ensure programme success.^27^

*Employee involvement* is another critical TQM principle. A lack of employee engagement in decision-making might affect the meal quality offered to students due to the low motivation of kitchen staff. TQM adaptation in the school nutrition programmes in Brazil and the United States highlights critical problems relating to employee participation within the TQM framework. In Brazil, a study revealed that the insufficient engagement of food service staff has caused low morale, leading to inconsistent meal quality.^28^ Another study in Kenya by Ogutu et al. indicates that low staff morale and engagement in school meal programmes affect service delivery.^29^ Similarly, Barnabas et al. hold that involving kitchen staff in menu planning increases student meal acceptance.^30^ These studies highlighted that advocating for regular staff meetings to encourage participation and ownership of the nutrition programme and implementing structured feedback mechanisms would improve meal quality for students.^31,32^

Improving food nutritional programmes requires continuous improvement and training. However, this has compromised meal quality in most countries. The TQM continuous improvement and training principle has been applied to improve school nutrition programmes and educational quality. In China, Chen et al. noted gaps in training for school food service personnel, affecting meal quality and nutritional standards.^33^ In a Russian study, Mohammadpour et al. indicated that inconsistent training for staff causes poor meal quality and low nutrition.^34^ Another study in Kenya by Ogutu et al. found that inadequate training for food service staff causes poor meal quality and low nutritional standards.^29^ Also, Ukpore in Nigeria emphasised that a lack of systematic evaluation processes hinders schools’ ability to adapt nutrition policies effectively.^35^ In improving the implementation of food nutrition, there is a need to support regular monitoring and feedback systems involving staff and students in the improvement process.^1,22^

## Materials and methods

This systematic review uses South Africa as a case study and document analysis. The study is registered at PROSPERO with the registration number 105607. Systematic reviews synthesise the current knowledge in a particular field. They address questions that cannot be answered by individual studies, identify issues in primary research that need to be corrected in future investigations, and generate or evaluate theories regarding how or why certain phenomena occur.^36^ A case study, on the other hand, is an in-depth investigation of a case that the researcher generates, usually one or more individuals or a programme, event, activity, or process.^37,38^ In addition, document analysis is a research method for systematically evaluating written or visual materials to extract meaningful information and insights.^39^ A structured approach to conducting document analysis includes eliciting themes based on the following: defining research objectives, selecting documents, familiarisation, coding or categorisation, data extraction, data organisation, analysing the extracted data to identify patterns, interpreting the findings, and contextualisation.^39,40^

Documents published between January 2000 and May 2024 were accessed. We utilised the Preferred Reporting Items for Systematic Reviews and Meta-Analyses (PRISMA) guidelines for reporting systematic reviews.^36^ The filled-in PRISMA 2020 27-item checklist is provided as a Supplemental file.^36^

The first and second authors were the assessors of the articles’ eligibility. Both of them screened the abstracts and full-text for ensuring eligibility, while the third and fourth authors drafted the review report and prepared the manuscript. Among all authors, there were no significant conflicting assessment results or ideas in the process of selecting eligible articles and reporting our study.

**Figure 1.**
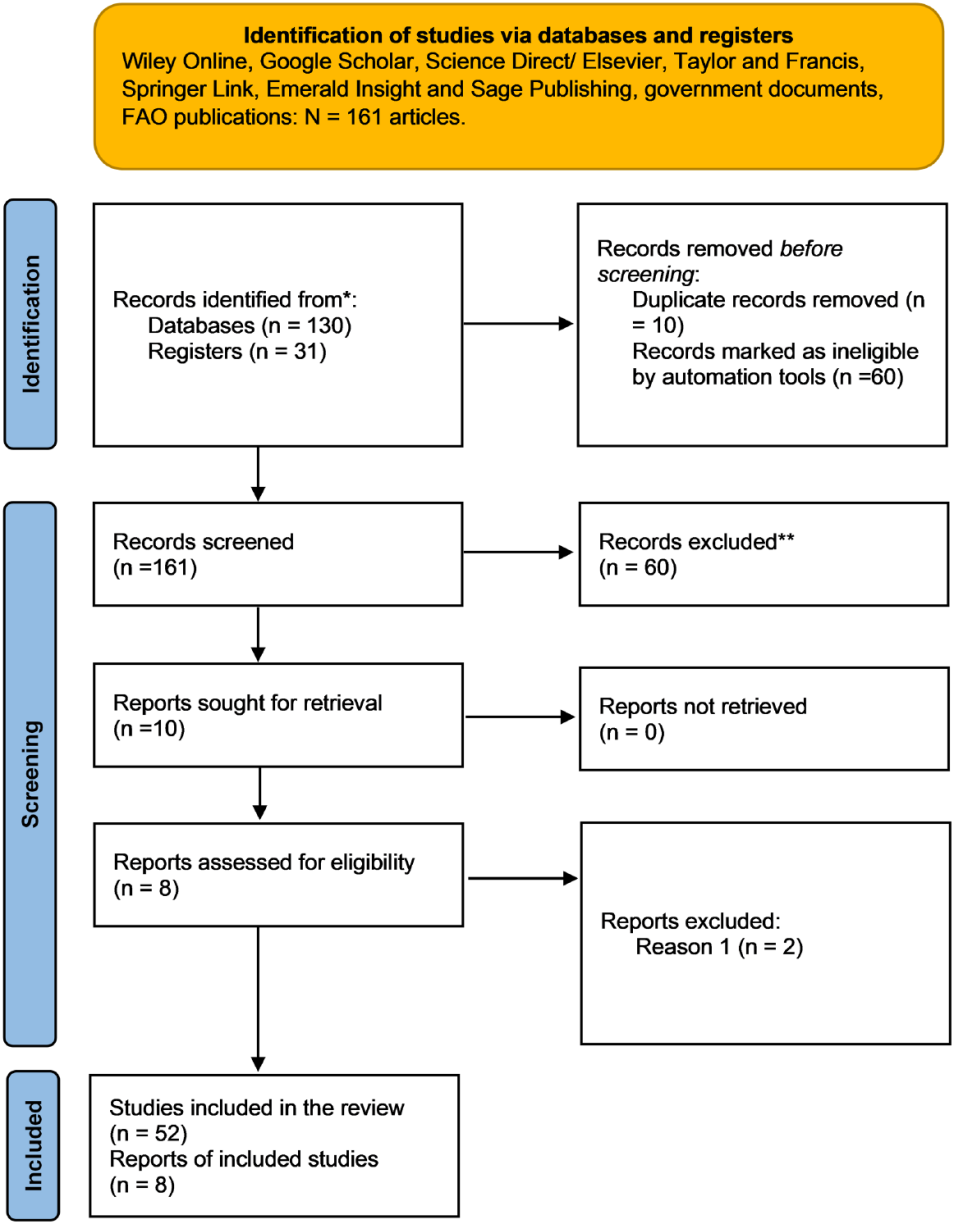
Literature search flow diagram.

The inclusion criteria of study materials were TQM relevance, the validity, and usefulness of studies, as illustrated in Figure 1 of the literature search flow diagram.

Figure 1 shows the literature search flow diagram to find the study materials for further analysis to address the study objective. The review started on 1 March 2024 and ended on 30 June 2024.

## Findings and discussion

This section presents the challenges of implementing NSNP’s TQM in South Africa. Table 1 summarises the findings.

**Table 1.** TQM implementation challenges in NSNP.

Main findings	Summary
Customer focus	- Failure to address the needs of students in quintiles 4 and 5, which are mostly urban areas, due to inadequate resources - Lack of funding from the Department of Basic Education (DBE) to accommodate increased enrolment. - Insufficient kitchen facilities - Inadequate stakeholder training - Late payments to food suppliers - Lack of targeted nutritional provisions for students in various grades
Employee Involvement	- Role ambiguity due to a lack of communication, and at times, the roles of the NSNP players are not clearly defined - The employment contract offers very little job security - Food handlers are not consulted in decision-making
Process-centric approaches	- Lack of school-level infrastructure to store and cook for students - Failure to establish standardised food portions - Lack of stakeholder consultations
Continuous improvement and training	- Nutrition coordinators’ - Excessive workload, - Lack of training, - Inadequate food delivery - Lack of an education curriculum on nutrition education
Relationship management	- Unregulated operation of tuck shops and general vendor systems can undermine healthy eating initiatives and compromise learner nutrition. - Lack of coordination of the stakeholders on funding, political landscape, nutrition policies, community involvement, and programme adaptability

Source: Authors.

## Customer focus

The role of organisations is to satisfy their customers or clients by providing high-quality products and services; customer focus is the first quality management concept to be considered. Thus, the final product must satisfy the consumer’s requirements and needs. The NSNP is intended to ‘improve the health and nutritional status of South African primary and high school students in rural areas (Quintile 1–3), to improve levels of school attendance, and to improve the learning capacity of children, which should, in turn, provide equal opportunities for the poor in terms of access to education’.^41,42^

One of the challenges is addressing the needs of students in quintiles 4 and 5, which are mostly urban areas, due to inadequate resources.^1^ Effective implementation of this programme and successful outreach to the beneficiaries can be complex and challenging, often hindered by logistical, socioeconomic, and systemic barriers that require innovative solutions and strategic partnerships to overcome.^43^ A study by Ferrero et al. and Hariram et al. indicates that the school nutrition programme is affected by the logistics process and inadequate funding.^19,44^ Likewise, Jean de Dieu et al. argued that the feeding programme failed to achieve the desired goals because the food supply was inconsistent^45^

The other challenge arises when schools enrol more students without increasing funding to accommodate the larger student body.’^2^ This is further strained by a ‘lack of a balanced meal and food shortages’ hampering the achievement of NSNP’s goals.’ It is important to ensure that enrolment is increased in the same proportion as resources to support students.^2^ Sidaner et al. concurred that Brazil’s national standards of menu composition were failing to meet consumer satisfaction due to inconsistencies in supply by farmers to meet demand.^46^ The findings of these studies indicate that governments are failing to satisfy customers by failing to support them financially in proportion to enrolment.

Mafugu identified factors such as insufficient kitchen facilities, inadequate stakeholder training and late payments to food suppliers.^10^ A study by Reardon et al. indicates that cases of obesity are rising in schools, and South Africa is not an exception.^47^ In South Africa, historically disadvantaged schools struggle with policy implementation, necessitating a customised TQM framework.^48^ A study of the NSNP in Mpumalanga, South Africa, revealed significant challenges in food safety management, particularly in rural schools lacking basic resources like electricity and potable water.^49^ However, the successful implementation of TQM principles in a South African primary school demonstrated improvements in various areas, including physical resources, curriculum, and financial management.^1,50^

The other common challenge is to emphasise meeting the nutritional needs of students, recognising that a ‘one-size fits all’ approach is inadequate. South African children are particularly vulnerable to the insidious effects of hidden hunger. It is recommended to optimise staple foods or crops by the biofortification process, besides supplementation and commercial fortification, to prevent nutrient deficiencies (vitamin A, iron, and zinc) by genetic manipulation, for instance.^51^ The government should seek partners or farmers who can supply food. This has been introduced in the Russian Federation, where the national feeding programme is tenderised or contracted to private suppliers who are expected to supply food accordingly to guarantee the programme’s success.^21^ Likewise, Ofori et al. explained that nutritional education needs integration with NSNP to improve the consumption of biofortified staple foods, and a supplementary government budget to improve the quality and quantity of the meals served to students.^52^ However, Africa is experiencing a double burden of malnutrition. There is a need for a national nutrition policy to nurture beneficial food processing and reduce unhealthy ultra-processed intake in Africa’s food system to maintain the standardised menu of NSNP in South Africa.

In addition, South Africa has made progress in implementing local food procurement for school feeding, which is beneficial for both students and local farmers. This progress is shown by successful local food sourcing for NSNP, promotion of dietary diversification among pupils and communities, and strengthening smallholder farmers’ capacity in local food procurement.^53^ Recent achievements in the South African NSNP include the continuous provision of cooked meals to primary and secondary students funded by the government, improved protein on the menu with the addition of canned pilchards and ultra-high temperature (UHT) treated milk, and deworming of all primary school students linked to feeding.^54^

In terms of local supply sourcing, the presence of school gardens is another significant challenge. In South Africa, the school garden programme is not uniformly implemented, despite the potency of school gardens to supply fresh, healthy, and nutritious foods. The concept of school garden, together with the school kitchen and a neighbouring vacant wild site, constitutes the ‘biodiverse edible schools’ concept, linking local urban nature and healthy food.^55^ A qualitative case study proved that the presence of school gardens is not beneficial for school communities only, but in terms of supporting sustainable food security, school gardens may be beneficial for students’ families and the broader community.^56^ Another study proved that school gardens improved academic performance, especially in the subjects of science and math, and dietary outcomes, especially fruit and vegetable consumption.^57^ There is a need to develop systems, such as solar borehole irrigation systems, to support school gardens at each school in South Africa. This will reduce the NSNP cost burden and make resources available to other educational needs^58^

In addition, collaborations among schools and universities offering Agricultural Study Programmes are recommended to support the establishment of school gardens. From the university perspective, school gardens can be a method for scaling up sustainable agriculture technologies through sustainable intensification technologies, showing a pathway to scaling agricultural innovations, and facilitating research-based agricultural curriculum in schools.^59^ This university-school collaboration may also provide opportunities for co-learning among university students and school students. School gardens can serve as valuable tools for sharing knowledge with the broader community. They can involve parents in the learning process and the maintenance of the gardens. Additionally, they can engage stakeholders in collaboration and foster commitment from the government or other organisations for financial support in establishing and maintaining the gardens.^59^

## Employee involvement

Organisations looking to implement TQM should strive to create a culture where workers believe they can make a difference. They share responsibility with management for assessing output quality at their assigned tiers. Here, effective internal communication plays a critical role in bolstering morale.

The first problem confronted is role ambiguity due to a lack of communication, and at times, the roles of the NSNP players are not clearly defined. The management process and information analysis are found to reduce role conflict, while a human resource focus may increase it.^60^ There is a need for organisations to ensure that roles are clearly stated to reduce food management. NSNP is involved in budgeting and managing allocated funds.^2^ However, food handling and allocation are done by another department. According to Mindzaeva and Arinushkina, the Russian Federation developed informatisation management and this improved internal school control systems.^61^ This is a successful story which has improved food distribution and reduced role ambiguity.

The additional challenge is that the employment contract offers very little job security. Food handlers face many challenges at the workplace: They can be replaced before the contract lapses if the School Governing Body yields to pressure from waiting candidates.^1^ In Brazil, a systematic review of the National School Feeding Programme quality indicators revealed gaps in methodology and the validity of evidence.^62^ A Nepalese study found that TQM practices positively influenced employee satisfaction and performance in technical institutions.^63^ In South Africa, research on secondary schools explored TQM as a management strategy to improve effectiveness, focussing on leadership and people management.^48^ These studies demonstrate the potential of TQM in education across different contexts, highlighting its impact on various aspects of school management, from resource allocation to employee satisfaction and overall effectiveness.

The South African NSNP is one great example of massive community and employee involvement contributing to its success. For instance, more than 50 thousand volunteer food handlers prepare nutritious meals for more than 9 million students, which meet the nutritional standards and address the students’ needs.^5^ Second, the important roles of the NSNP school coordinator include managing and supervising the NSNP’s daily activities and ensuring that the guidelines have been executed adequately. Third, all NSNP stakeholders actively participated in capacity-building workshops to improve their skills and knowledge relevant to NSNP implementation. Collaboration with government departments and Non-Governmental Organisations is built in a suitable manner to promote nutrition education, food safety, and other initiatives related to NSNP, for example, school gardens and deworming programmes.^1^ The challenges are ensuring consistent volunteer participation over time and providing ongoing training and support to volunteers and the NSNP school coordinator.

## Process-centric approaches

The other challenge was the lack of school-level infrastructure to store and cook for students. Evidence on evaluations of the NSNP highlights the inadequacy of school-level infrastructure.^1^ Mafugu add that the nutrient content of NSNP meals varies due to long storage periods or exposure to light and oxygen, highlighting the importance of proper storage, stock rotation and timely use.^10^ Lack of infrastructure impacts several NSNP processes. ‘The conditions of the volunteer food handlers (VHFs) are difficult; they work on average 6 h of hard labour, and in some cases, in very terrible conditions, with no running water or even a suitable kitchen’. The lack of volunteer food handlers is a common problem in KwaZulu-Natal, Mpumalanga and Limpopo. Inadequate facilities and a lack of necessary utensils for food preparation and storage are issues in Gauteng, Mpumalanga, KwaZulu-Natal, the Western Cape, and the Eastern Cape. Consequently, Frodeman and Holbrook draws attention to the National Science Foundation’s battle to balance public responsibility and scientific autonomy, which is connected to the difficulty of providing leadership in TQM.^64^ One possible answer to this problem is the necessity of strategic, stratified task teams.^65^ Quality must be built into every process step to ensure that the students who benefit from the NSNP receive nutritious meals. Appointing service providers, monitoring and supporting programmes, and bolstering district and school-level capacity are all within the purview of provincial-level bureaucrats, while monitoring and supporting programmes at the school level fall under the ambit of district-level bureaucrats.^42^

In addition, poor road infrastructure is another significant challenge, especially in rural areas of South Africa. Drawing from Ondo State, Nigeria, poor rural road infrastructure was to blame was low agricultural productivity and food insecurity.^66^ During the rainy season, poor road infrastructure leads to poor access, dampens connectivity between rural and urban areas, and delays deliveries of school meal supplies.^67^ Bad access also made rural farmers find difficulties in accessing urban markets, decreasing trade and other poverty reduction efforts.^67^ The rural transport infrastructure sector is a critical force for sustainable development that is interwoven with many other sectors. There is a need for intergovernmental collaboration in formulating strategies and efforts to improve rural road infrastructure, additional integration of the sectors, and increased usage of systems approaches, viewing rural road infrastructure as an active part of many other sectors and a key leverage point within rural development as a whole.^68^

The other challenge is the failure to establish standardised food portions. In school nutrition, a study of South African dietitians revealed that while efforts were made to maximise nutrient preservation, systematic documentation was lacking.^69^ An analysis of meals served under the South African NSNP found that many meals did not meet nutrient standards for energy, carbohydrates, and certain micronutrients, particularly for older students.^42^ There is a need for improved quality management in school nutrition programmes, including better food preparation procedures and nutrient preservation techniques.^70^ It is also important to standardise the health behaviour of all individuals who are responsible for food preparation, such as hand-washing procedures.

## Continuous improvement and training

The other challenge in TQM implementation was a lack of training, which affected food delivery. According to Mawela and van den Berg, school principals and nutrition coordinators are well-informed about the programme’s management roles and responsibilities; however, they lack some of the essential food handling skills.^2^ Workshops and seminars on budgeting, sustainable agriculture, and meal planning are essential for the continuous improvement of the NSNP programme.^1^

The South African Department of Basic Education is faced with a lack of an education curriculum on nutrition education.^71^ Efforts to improve quality should be continuous. For the TQM strategy to succeed, individuals at all levels must consistently stay vigilant and conduct quality checks. In Brazil, the National School Feeding Programme or the Programa Nacional de Alimentaço Escolar, aims to enhance children’s nutritional status and promote healthy eating habits through meal provision and nutrition education.^72^ A case study in Rio de Janeiro demonstrated that empowerment-based curriculum development involving community stakeholders can increase commitment to implementation.^73^ In South Africa, TQM has been proposed as a potential solution to education system challenges, emphasising customer focus and continuous improvement.^74^ Another study in South Korea revealed that while school dieticians strive to maximise nutrient preservation, systematic documentation and quality control in food production need improvement.^69^

While there is no evidence yet of school gardens becoming part of the core curriculum or extracurricular activities at school, the gardens offer students the opportunity to increase their more formal knowledge about agricultural production. The work in school food gardens should become part of the curriculum. It is crucial to review the food curriculum, connect it to school meals, and refocus on monitoring and reporting school food standards.^75^ It is also necessary to include family farming in the school feeding programme. This initiative needs government support for community-based projects and contract farming. The champion of this initiative is the Brazilian home-grown school feeding approach, which stimulates local agricultural and economic development. The approach includes procurement from small-scale black farmers, supporting them financially.^76^ Studies show that the absence of locally grown foods negatively impacts the quality of school meals, making them less healthy and culturally inappropriate.^76^ The inclusion of local foods in the schools meal plan was associated with higher consumption of fruits and vegetables and lower consumption of processed foods among students, indicating a positive relationship between locally grown foods and fruit and vegetable consumption^77^

Continuous improvement is needed in all aspects of NSNP execution. The school lockdown during the COVID-19 outbreak led to many innovations and improvements in the food delivery aspect of school nutrition programmes in the United States, and continue to be implemented after the pandemic.^78^ However, implementing TQM in schools requires rigorous effort and openness to rethinking problems.^74^ In the context of BRIC countries (Brazil, Russia, India, and China), TQM and strategic process improvement are still developing, presenting both opportunities and challenges for practitioners in these emerging economies.^79,80^

The suggested continuous improvement and training activities include team-building activities, seminars with industry experts and classroom training.^80^ TQM practices were found to be partially correlated with quality performance, with quality culture being the dominant practice.^81^ Other practices like quality systems, training, teamwork, and benchmarking also showed positive relationships with performance. To implement TQM in schools, a management strategy involving principals, educators, students, and parents is necessary^81^

Communication, employee involvement, and process orientation can be identified when implementing, monitoring, and evaluating the programme. In terms of system integration, ‘the National School Feeding Programme or the Programa Nacional de Alimentaço Escolar is an excellent example of integrated nutrition that strives to be universal in many respects by supplying meals for nursery, school-aged, and even adult basic education students throughout Brazil, while allowing for geographical and cultural specificity’.^21,46^ Regular training for food handlers ensures hygienic preparation and storage practices. In addition, certificates of Acceptability issued by municipalities validate compliance. Empowering NSNP school coordinators with training and support enhances programme implementation.^1^

## Relationship management

The challenge confronting the effective implementation of the NSNP is the unregulated operation of tuckshops and general vendor systems that can undermine healthy eating initiatives and compromise learner nutrition. The NSNP contributes significantly to food security and academic output in South African schools but requires reengineering to control non-communicable disease risk factors.^78,82^ Implementation challenges in South African schools include inadequate budgets, untrained food providers, and the prevalence of low-nutritional-value foods from tuck shops and vendors.^10^ TQM in schools represents a shift from traditional bureaucratic management towards empowerment and participation, potentially improving school effectiveness.^48,82,83^

Managers need to develop mechanisms for imparting a supportive work environment to foster healthy exchange relationships with people under them, the employees, resulting in employee retention.^84,85^ In the school meals programme, the relationship between the programme managers, employees, and student needs should be maintained to ensure adequate TQM implementation in NSNP. Miscommunication and bad relationships between parties may lead to adverse effects of TQM implementation in NSNP. NSNP successfully builds stakeholder engagement and collaboration, such as with the school communities and the local authorities.

Relationship management is at the heart of the NSNP’s success. Maintaining positive relationships over time requires consistent effort. Understanding local customs and traditions helps build respectful relationships because South Africa’s diverse cultural landscape demands sensitivity. Effective conflict resolution mechanisms ensure that relationships remain constructive.

## Policy recommendations

Policies to ensure the success of the NSNP should promote teachers’ and instructors’ cooperation to improve students’ grades and overall performance. All stakeholders must demonstrate unwavering dedication to the school’s mission. Workers who prepare and serve students’ meals should be instructed on their responsibilities in the classroom and the importance of teamwork, and teachers will benefit from being equipped with leadership abilities and enthusiasm. NSNPs at the school level can significantly benefit from fostering a culture of collaboration among personnel. Principals should be involved in their school’s efforts to meet the Department of Basic Education requirements by coordinating efforts among teachers and staff and monitoring the National School Lunch Programme. Lessons can be drawn from India, which, through the Department of School Education and Literacy, prescribed a comprehensive and elaborate mechanism for monitoring and supervision of the Mid-Day Meal Scheme, which included local-level monitoring with local representatives and mothers’ committees to monitor the programme’s implementation daily, in critical areas of hygiene and cleanliness in cooking and serving; timeliness in the procurement of resources, and implementation of varied menus.

There is a critical need for informed and context-specific procurement models within Home-Grown School Feeding (HGSF) approaches that intentionally link school meal demand to local smallholder agricultural production, such as the Brazilian National School Feeding Programme. Such models can stimulate local agricultural growth, create market access opportunities for small-scale farmers, and contribute to broader rural economic development while ensuring sustainable and nutritious food provision for learners.

Well-designed monitoring systems must be implemented to ensure that the required quality and quantity of food is delivered to the students and to monitor the supply, storage and preparation of food and general food hygiene. This could increase the likelihood of improved nutrition for students. Schools should emphasise food safety, as providing unsafe food to students is counterproductive. Following Brazil’s National School Feeding Programme, local food outsourcing from farmers has reduced reliance on ultra-processed foods, a significant challenge in South Africa, as procurement of ultra-processed food, such as tinned fish, is popular.

The targeting system (the quintile system) needs to be reviewed by looking at the school’s location and the available resources, and considering individual students’ social context, for example, their household income and living environment, which would guarantee that a larger number of poor students will benefit from the programme. Furthermore, provision should be made for an unexpected increase in enrolment, as has been the case at most schools, which has led to some students not receiving meals. Lessons can be drawn from Russia, who, through the Department of Social Nutrition, raise awareness of the importance of healthy school lunches; the Health of Our Children, a Russian Non-Government Organisation, organises Milk Day, milk lessons, contests, and festivals at national and provincial levels. School Milk’s website serves as a hub for communication between various parties. In addition, the Chinese Nutrition Improvement Programme distributes about four Yuan (0.65US$) per student per school day, which is paid directly to schools to improve or rebuild their cafeterias and provide free meals (mainly lunch). The programme includes a nutrition education component for students, parents, school staff, and caterers.

Overall, current challenges can be overcome through government support, awareness of cultural and environmental influences, and the creation of clear, valuable guidelines and assistance for better implementation of TQM in NSNP. The South African government should work on creating a national nutritional guideline that includes a standard menu and suggestions for optimising school menus. This guideline should be mandatory for all schools. There is also a need to formulate strategies to extend the feeding modalities, such as the addition of school snacks and take-home rations. It is also recommended to employ digital management tools to improve the internal control systems for quality assurance within schools, implementing TQM continuously. In addition, collaborations among the following government departments, such as the Department of Basic Education, the Department of Agriculture, Water and Sanitation, Health, and Transport, are needed to ensure effective implementation of TQM in the South African NSNP.

## Strengths and limitations of the study

Several strengths are identified regarding this study. The study utilised a qualitative case study design, which provides an in-depth understanding of the challenges faced in implementing TQM in the South African NSNP. This approach allows for a comprehensive exploration of various factors affecting the programme, making it more insightful. Second, the study uses document analysis, which adds rigour to the research by evaluating primary data policy documents, programme reports, and other literature through the PRISMA systematic review methodology. This method ensures a structured data collection approach and enhances the credibility of the findings. Third, the thematic analysis helped to identify several critical challenges related to different aspects of the NSNP. Finally, the study addresses the challenges faced in rural areas, which are often more marginalised and lack resources. This helps to identify regional disparities in the implementation of the NSNP and ensures that the needs of the most disadvantaged communities are considered.

This study was not without limitations. One notable limitation is that this study used document analysis as the primary material to reveal the challenges of implementing TQM aspects in the South African NSNP. This study material cannot fully capture all the challenges experienced by the programme executors in schools. Thus, a qualitative study utilising the in-depth interview method is needed to fully reveal these challenges. Participants may come from various stakeholders, such as students, teachers, parents, and the cooking personnel. There is also a need for a national study conducted in South Africa among these stakeholders to provide more insights into the practical challenges on a day-to-day basis.

## Conclusion

The study aims to explore challenges in implementing TQM in the South African NSNP. A case study research methodology was used in this study. The findings were generated from documents in the public domain extracted through the PRISMA method, and the records were analysed using thematic analysis. The Ecological Systems Theory, guided the study, developed by Urie Bronfenbrenner, emphasising the influence of multiple environmental systems on an individual’s development. The study concludes that schools should enrol students who match the available resources for food. Additionally, the resources are overstretched because there are more students than available resources. This study found a limitation on participation in decision-making, making it challenging to implement the food programme since stakeholders lack ownership of the programme. Also, some schools in South Africa lack appropriate kitchen infrastructure, making it a challenge to store food in the right conditions. This indicates that appropriate kitchens for cooking the food are of substandard quality, making it difficult to achieve the desired results. To address this, there is a need to improve the training that stakeholders, including students, need to be educated, so that nutrition programmes should be included in the education curriculum.

## Supplemental Material

sj-docx-1-phj-10.1177_22799036251380781 – Supplemental material for Transforming South Africa’s school nutrition programme for educational success: A review on challenges and prospects Supplemental material, sj-docx-1-phj-10.1177_22799036251380781 for Transforming South Africa’s school nutrition programme for educational success: A review on challenges and prospects by Adrino Mazenda, Aboleleng Monedi, Ni Putu Wulan Purnama Sari and Chamunorwa Huni in Journal of Public Health Research
